# Microbiota and IL-33/31 Axis Linkage: Implications and Therapeutic Perspectives in Atopic Dermatitis and Psoriasis

**DOI:** 10.3390/biom13071100

**Published:** 2023-07-10

**Authors:** Laura Bonzano, Francesco Borgia, Rossella Casella, Andrea Miniello, Eustachio Nettis, Sebastiano Gangemi

**Affiliations:** 1 Dermatology Unit, Azienda Unità Sanitaria Locale-IRCCS di Reggio Emilia, 42122 Reggio Emilia, Italy; laura.bonzano@ausl.re.it; 2 Department of Clinical and Experimental Medicine, Section of Dermatology, University of Messina, 98122 Messina, Italy; 3 Department of Emergency and Organ Transplantation, School of Allergology and Clinical Immunology, University of Bari Aldo Moro, Policlinico di Bari, 70120 Bari, Italy; rossellacasella7@gmail.com (R.C.); miniello_andrea@yahoo.it (A.M.); ambulatorio.allergologia@uniba.it (E.N.); 4 School and Division of Allergy and Clinical Immunology, Department of Clinical and Experimental Medicine, University of Messina, 98122 Messina, Italy; sebastiano.gangemi@unime.it

**Keywords:** microbiota, IL-33, IL-31, cytokines, psoriasis, atopic dermatitis, inflammation, skin, pathogenesis, treatment

## Abstract

Microbiome dysbiosis and cytokine alternations are key features of atopic dermatitis (AD) and psoriasis (PsO), two of the most prevalent and burdensome pruritic skin conditions worldwide. Interleukin (IL)-33 and IL-31 have been recognized to be major players who act synergistically in the pathogenesis and maintenance of different chronic inflammatory conditions and pruritic skin disorders, including AD and PsO, and their potential role as therapeutic targets is being thoroughly investigated. The bidirectional interplay between dysbiosis and immunological changes has been extensively studied, but there is still debate regarding which of these two factors is the actual causative culprit behind the aetiopathological process that ultimately leads to AD and PsO. We conducted a literature review on the Pubmed database assessing articles of immunology, dermatology, microbiology and allergology with the aim to strengthen the hypothesis that dysbiosis is at the origin of the IL-33/IL-31 dysregulation that contributes to the pathogenesis of AD and PsO. Finally, we discussed the therapeutic options currently in development for the treatment of these skin conditions targeting IL-31, IL-33 and/or the microbiome.

## 1. Introduction

Both cytokine networks and microbiota have been the subject of ever-growing interest in the field of dermatology as biomarkers and possible therapeutic targets for skin diseases with major worldwide social and economic burden, such as psoriasis and atopic dermatitis (AD).

Dysbiosis has been recognized as a hallmark of AD for many decades, while interest in the role of the microbiota in psoriasis and other cutaneous diseases has been sparked only more recently. Still, despite the massive literature available addressing the cytokine and microbial changes in these diseases, the bidirectional relationship between dysbiosis and chronic inflammation is far from being exhaustively uncovered.

Interleukin (IL)-31 and IL-33 are both crucial players in the development of AD, and studies are being conducted to elucidate their role in psoriasis. Furthermore, their synergistic activity has recently led to the conception of the “IL-33/IL-31 axis” hypothesis, which has been shown to be involved in the pathogenesis of various chronic inflammatory conditions, including AD.

### 1.1. Overview on Atopic Dermatitis

Atopic dermatitis is the most common chronic inflammatory skin condition. It is defined by the presence of pruritus and eczema (with age-specific patterns), and it can be associated with lesions such as xerosis, exudate, keratosis pilaris, pityriasis alba, hyperlinear palms, ichthyosis, lichenificaction or periorbital changes. It is strongly associated with atopy and commonly coexists with other allergic conditions. It manifests with a chronic or relapsing history and usually has an onset at an early age (typically in the first 2 years of life). The diagnosis of AD is strictly clinical and is based on evaluation of family and patient history, morphology and distribution of skin lesions. Diagnostic sets of criteria have been developed by different research groups, and the most used ones in the literature are the Hanifin–Rajka criteria and the United Kingdom Working Party group criteria [1]. Various physician-reported and patient-reported tools have been validated for the evaluation of AD lesions, symptom control and quality of life [2,3,4]. The aetiopathogenesis is multifactorial. Type-2 inflammatory mechanisms are typical of the disease, but there is increasing evidence that the disorder involves multiple immune pathways. Mutations or polymorphisms of the FLG (filaggrin) gene are well-known risk factors for the development of AD and lead to the impairment of skin integrity (affecting physiologic functions such as hydration, pH, temperature, elasticity and melanin content), but other genes associated with other endotypes of AD have been investigated as well [5,6]. Skin hygiene practices and emollient therapy are the essence of AD treatment. Topical anti-inflammatory agents (corticosteroids or calcineurin inhibitors) are the first-line therapy in patients who failed to respond to good skin care and correct use of moisturizers alone. Systemic immunosuppressive agents (e.g., cyclosporine, azathioprine, methotrexate), monoclonal antibodies (dupilumab, tralokinumab) or JAK inhibitors (baricitinib, upadacitinib, abrocitinib) can be employed in those cases which are resistant to optimized topical regimens and/or phototherapy [7,8,9,10]. Despite the therapeutic progresses made in the last decade with the creation of innovative targeted therapies, AD remains a disease with no cure and with a massive impact on the economy and the quality of life of patients, their families and caregivers, and it is currently the leading cause of global burden from skin diseases [11,12].

### 1.2. Overview on Psoriasis

Psoriasis is a chronic immune-mediated inflammatory skin disease, characterized by symmetrical well-demarcated erythematous plaques covered by silvery-white scales [13]. Like in the case of AD, psoriatic skin function is severely impaired [14]. It affects around 2% of the population worldwide. The prevalence of psoriasis depends on ethnicity, genetic background, ageing and environmental factors (e.g., stress, infections, trauma, sun exposure) [15]. The pathogenesis is multifactorial, involving dysregulated inflammation and genetic associations [16]; in particular, psoriasis susceptibility 1 (PSORS1) is a major susceptibility locus and the allele HLA-Cw6 is associated with early and acute onset. Dendritic cells, activated by antimicrobial peptides (AMPs) (e.g., LL37, β-defensins, S100), which are secreted by damaged keratinocytes, play a key role in the development of the psoriatic plaque [13]. T cells interact with dendritic cells and macrophages, partly mediated by their secreted cytokines (e.g., type I INF, tumour necrosis factor (TNF), interleukin (IL)-17, IL-23, IL-36, IL-12) [15]. In particular, these cytokines are implicated in Th1 and Th17 differentiation and proliferation [13]. This complex interaction between the innate and adaptive immune system results in skin inflammation and aberrant hyperproliferation of keratinocytes [17]. There are several subtypes of psoriasis, but the plaque-type is the most common [18]. Recent studies identified that psoriatic skin inflammation causes tissue and organ dysfunction [19]; indeed, it is associated with psoriatic arthropathy, cardiovascular, diabetes mellitus, obesity, inflammatory bowel disease and hepatic comorbidities [13,18]. The diagnosis is primarily clinical. First-line treatment involves topical therapies including corticosteroids and vitamin D3 analogues and/or phototherapy. Patients with more severe and refractory symptoms might require systemic therapy with conventional systemic agents (e.g., methotrexate and ciclosporin) or targeted biologics (TNF, IL-17 and IL-23 inhibitors) and oral small molecule inhibitors (dimethyl fumarate and apremilast) [18].

### 1.3. The IL-33/IL-31 Axis

Although these diseases are characterized by different phenotypes, they often share common characteristics in their inflammatory milieu, such as the overexpression of IL-31 and IL-33.

IL-31, a member of the gp130/IL-6 family with an anti-parallel four-helix bundle structure, plays a key role in inducing pruritus in different diseases, such as atopic [20,21] and contact dermatitis [22], psoriasis [23] and chronic urticaria [24]. It is produced by activated Th2 cells, but also mast cells (MCs), macrophages, dendritic cells (DCs), eosinophils and basophils can express IL-31 to a lower extent [25]. Its main targets are fibroblasts and eosinophils, which are activated through IL-31 receptor (IL-31R), a heterodimeric complex composed of two subunits, IL-31 receptor alpha (IL-31RA) and oncostatin M receptor B (OSMRβ). Four isoforms of IL-31RA were identified (IL-31RA-v1 to IL-31RA-v4) [26]. IL-31R is primarily expressed in nonhematopoietic tissue, in the skin and in the endothelium, suggesting that IL-31 has several functions in regulating these tissue responses, in stimulating pro-inflammatory cytokines, in regulating cell proliferation and in tissue remodelling. IL-31 acts through three signalling pathways: JAK/STAT (Janus-activated kinase/signal transducer and activator of transcription), PI3K/AKT (phosphatidylinositol 3-kinase/protein kinase) and MAPK (mitogen-activated protein kinase) pathways [27,28].

IL-33, a member of IL-1 family, is the second molecule of our interest. It performs various roles in tissue homeostasis, growth and repair, and it is constitutively expressed by endothelial cells, epithelial cells of barrier tissue, fibroblast-like cells and myofibroblasts as a full-length immature form [29,30,31]. Its expression can be induced in MCs and DCs in inflammatory skin diseases, such as AD, chronic urticaria and vitiligo [30,32]. IL-33 also plays a critical role in allergic diseases [26], inducing the activation of Th2 immunity and the production of IL-4, IL-5 and IL-13, the polarisation of macrophages and the degranulation of basophils and eosinophils [32]. Finally, IL-33 can be released by MCs after physiological stress, too, as occurs in psoriasis [33]. Similarly to IL-1α, it requires cleavage to increase its activity and has a dual role: IL-33 acts extracellularly as a cytokine, recruiting and activating immune cells upon necrosis and inflammation (alarmin function), and intracellularly as a nuclear factor, regulating gene expression and homeostasis [32]. IL-33 acts through different signalling pathways, such as JNK (c-Jun-N-terminal kinase), NF-κB (nuclear factor κB) and MAPK pathways [32,33].

Since IL-33 was first identified, many studies have suggested that these inflammatory patterns were connected, leading to the newest theory of an “IL-33/IL-31 axis” [29]. According to Di Salvo et al. [27], the presence of one interleukin might stimulate the induction of the other. When epithelial cells are exposed to an allergen, bacterium or virus, IL-33 is cleaved into mature forms by both an exogenous and endogenous proteolytic mechanism. Then, it binds to its receptor complex composed of ST2 and IL-1 receptor accessory protein (IL-1RAP), leading to the dimerisation of the IL-1RAP intracellular domain called TIR (toll/interleukin-1 receptor), which is necessary for the activation of many signalling pathways (e.g., MAPK, JNKs, NF-κB). In particular, the IL-33/NF-κB signalling is reported to induce the release of IL-31. In addition, IL-4, enhanced by IL-33, induces the IL-31 genetic expression [26,29,34,35]. Other studies showed a tight correlation between serum and tissue levels of IL-31 and IL-33 and the intensity of the symptoms and the severity of the signs, suggesting a close correlation between these two interleukins in many pathologies [31].

### 1.4. Microbiota, IL-33/IL-31 Axis and Their Pathogenetic Link with Skin Conditions

The skin is an ecosystem which harbours millions of bacteria, fungi and viruses that compose the skin microbiota and have pivotal roles in the maintenance of physiologic cutaneous homeostasis, protection against pathogens, education of the immune system, neuroendocrine regulation and breakdown and generation of bioproducts [36,37]. Numerous studies have also demonstrated a bidirectional connection between the skin and gut health and allostasis (gut–skin axis). Indeed, gastrointestinal disorders are often accompanied by cutaneous manifestations, and the gut microbiome seems to have a role in multiple chronic inflammatory conditions [38,39,40]. Extensive literature has been published regarding the microbial alterations observed in AD and psoriasis and the role of skin and gut dysbiosis in the development of cutaneous diseases has gained noticeable interest in the last decade [40,41,42,43,44,45,46]. A variety of interventions that can alter the composition or functional capacity of the microbiome have been investigated as therapeutic or preventative strategies for both AD and psoriasis, including prebiotics, probiotics, [47,48,49,50,51,52,53,54] antibiotic exposure avoidance [55,56,57,58,59,60], diets [61,62,63,64], faecal microbial transplantation [65,66,67] and topical bacteriotherapy [68]. Moreover, recent findings highlight the possibility to restore global eubiosis (along with skin function) in AD by blocking IL-4Rα signalling with biological drugs [69,70]. Nevertheless, the causal relationship between dysbiosis and AD or psoriasis remains unclear, and the molecular mechanisms by which changes in the microbiome lead to a chronic inflammatory response (or vice versa) in these diseases are still being investigated [40,71,72]. IL-33 has already been found to drive multiple scenarios of dysbiosis-associated chronic inflammation, including gastrointestinal [73,74], dental [75,76,77], allergologic [78,79,80], pulmonary [81] and oncologic [82] conditions, and a previous study by Murdaca et al. reviewed the role of the IL-33/IL-31 axis in the development of autoimmune and allergic disorders, including AD [29].

The aim of this review is to delve into the link between dysbiosis and the IL-33/IL-31 axis in AD and psoriasis, supporting the idea that dysbiosis can act as an aetiological culprit in the development of skin conditions through immunodysregulation.

The bibliographic search was conducted using Pubmed. The keywords selected for our searching process were “dysbiosis”, “IL-31”, “IL-33”, “IL-33/31 axis” and “microbiota” combined with “atopic dermatitis” and “psoriasis”. In our review, we included all the research articles indexed in peer-reviewed scientific journals that reported the role of dysbiosis and the IL-33/31 axis in these diseases.

## 2. Dysbiosis and IL-33/31 Axis in Atopic Dermatitis and Psoriasis

### 2.1. Dysbiosis and IL-33/31 Axis: The Role in Atopic Dermatitis

Hundreds of studies investigating the aetiology of AD are conducted every year in order to engineer new therapeutic approaches for this disease. Dysbiosis has been recognized as a hallmark of AD for more than 50 years [83], and there is growing evidence regarding its role in the aetiopathogenesis of the disease [41]. Staphylococcus Aureus in particular has been one of the most studied microorganisms in AD: it is the most common cause of infection-induced flares [77,84], its prevalence in the lesions of AD patients is estimated to be 70% (and 62% in their nostrils) [85], its density and the presence of clonal S.aureus strains have been associated with disease severity [86,87] and studies on an infant population showed that S.aureus colonization precedes clinical diagnosis of AD [88,89].

**Figure 1:**
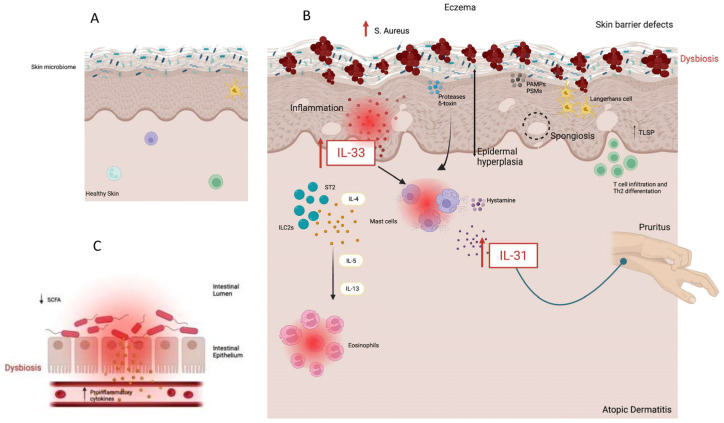
The difference between healthy skin (**A**) and atopic dermatitis (**B**). In picture (**B**), the role of *S. Aureus* in eliciting the production of IL-33 by keratinocytes is represented. Further, gut dysbiosis (**C**) also induces the inflammatory pathway.

Different staphylococcal factors can disrupt the epidermal barrier and induce programmed cell death with the subsequent release of inflammatory molecules [90], but cell death-independent IL-33 production induced by Staphylococcus has been observed as well. In a study investigating the regulation of IL-33 in the context of inflammation, *S. aureus* was found to be able to elicit IL-33 in keratinocytes after intradermal injection without overt signs of epidermal necrosis [91]. This finding was then further expanded by Al Kindi et al. [92], who studied seven different staphylococcal species and found out that only Staphylococcus aureus was able to trigger IL-33 and thymic stromal lymphopoietin (TSLP) release by human epithelial keratinocytes. Moreover, they identified *S. aureus* immunoglobulin-binding protein (Sbi) as the bioactive virulence factor responsible for the release of IL-33 (independently of cell death and toll-like receptor 2 recognition) by showing that loss-of-function Sbi2 S aureus mutants induced little or no type 2 immune activity. The authors theorized that Sbi could directly bind to the surface receptor on keratinocytes through recognition of an immunoglobulin domain to mediate the release of TSLP and IL-33 (Figure 1). Still, other studies showed that staphylococcal serine protease-like proteins (which were not able to elicit IL-33 release on human keratinocytes in Al Kindi et al.’s study) can induce a type 2 reaction in the airways [93,94,95], thus suggesting that molecular triggers for atopy can vary in different tissue. 

The role of other staphylococcal toxins in inflammatory disorders has been studied as well [71,96,97,98]. In particular, sublytic concentrations of α-toxin and Staphylococcus enterotoxin B (SEB) have been shown to significantly increase the expression of IL-31 and IL-31RA in peripheral blood mononuclear cells from AD patients compared to healthy patients [21,99,100]. IL-33 and ST2 expression are also upregulated by topical exposure to SEB in mouse AD skin [101]. A 2018 meta-analysis showed that the serum levels of anti-SEB IgEs are several times higher in AD patients compared to controls, but their presence could not be correlated with disease severity [102]. Staphylococcus δ-toxin can also directly induce mast cell degranulation (and release of IL-31) without mast cell lysis in an IgE-independent fashion via MRGPRX2 receptor binding [103,104,105]. Finally, studies on the gut microbiome of AD patients highlight a reduced capacity to produce short-chain fatty acids [106,107], which are renowned for their anti-inflammatory properties and their role in the maintenance of skin barrier function and have been demonstrated in vitro to suppress the growth of S. *aures* [108,109,110], thus suggesting a mutual interaction between the gut and the skin dysbiosis in atopic dermatitis [111]. Aside from Staphylococci, there are many other microbial species (including bacteria, viruses and fungi) which have been found to be dysregulated in the skin and gut of patients affected by AD [38,112,113,114,115]. A decrease in size and number of sebaceous glands and in the levels of sebum production have been noticed in AD patients compared to a healthy population [116], and a decreased abundance of lipophilic microbes such as Cutibacteria (whose abundance is significantly correlated with the level of skin sebum) [117] has also been noticed in patients affected by AD [44,118]. Based on these premises, Qiu et al. recently conducted a study where they explored the role of sebum and its related microbiota in AD. They found out that propionate, one of the most abundant sebum microbial metabolites, is significantly reduced in the skin of AD patients, and they tried to treat an MC903-induced AD-like mouse model [119] with a topical application of propionate. The results showed that propionate can attenuate AD by inhibiting IL-33 production without affecting the expression of other alarmins such as TLSP and IL-25. Attenuation of clinical signs was also observed after application of a mixture of Cutibacterium acnes and 2% glycerol [120].

### 2.2. Dysbiosis and IL-33/31 Axis: The Role in Psoriasis

Although the aetiology of psoriasis is still debated, several studies showed a correlation between the skin and gut microbiota dysbiosis and the occurrence of psoriasis [121,122,123,124], and the severity and status of the disease has been shown to be influenced by microbiota [125,126,127], which modifies the immunological and inflammatory response [126,128]. Yan D. et al. found a lower diversity and altered relative abundance for certain bacterial taxa in the skin and gut of psoriatic patients [129]. Fry and Baker suggested that the gut is the possible origin of the dysbiosis observed in psoriasis [130].

Sixty percent of people affected by psoriasis have their skin colonized by *S. aureus*, pointing out the possibility that it may exacerbate the skin condition and it may trigger an inflammatory Th17 response, inducing the perpetuation of keratinocyte proliferation [131]. S. pyogenes is also considered a trigger for the development and the exacerbations of psoriasis [131,132]. Firmicutes, Actinobacteria and Proteobacteria are the most prevalent phyla in both psoriatic lesions and healthy skin [133]. The most frequently reported findings in gut microbiome of psoriasis patients are the increase in *Firmicutes, Actinobacteria* and *Lachnospiraceae* and the reduction in *Bacteroides, Akkermansia* spp. and *Faecalibacterium prausnitizij* [134,135,136]. The reduction in the last one limits its anti-inflammatory effect in the gut and other organs through the production of short-chain fatty acids (SCFAs). In particular, butyrate, a major SCFA, limits the formation of reactive oxygen species, inhibits the adhesion, proliferation, translocation and production of cytokines by cells of the immune response, maintains the intestinal barrier integrity and blocks the NF-κB signalling pathway-mediated response and the production of IL-6 [44].

Among viruses, it seems that a human papillomavirus (HPV) infection of the keratinocyte induces epidermal hyperproliferation [130]; and among fungi, *Candida albicans* and *Malassezia* spp. colonization has been associated with exacerbations [136]. Malassezia is a common inhabitant of the scalp [129] and, according to Watanabe et al., *Malassezia sympodialis* can increase the production of the pro-inflammatory cytokines, such as TNF-α, IL-1, IL-6 and IL-8 in the skin, and stimulate keratinocyte proliferation [137]. Faecal samples of psoriatic patients showed an increased concentration of IL-1α, which stimulates the accumulation of T lymphocytes, activates the process of antigen presentation and is also involved in the process of stimulating Th-17 lymphocytes in the skin of patients. Therefore, increased expression of IL-1α [44] and other cytokines of its family [32,138], such as IL-33, in the intestinal lumen of patients with psoriasis may be the link between the gut inflammation accompanying this dermatosis and skin lesions [44]. As written before, the alarmin IL-33 is capable of activating both innate and adaptive immunity and, when it cooperates with IL-31, inducing the potential inflammatory pathway in allergic and inflammatory diseases [29]. Several studies showed increased serum levels of IL-33 in psoriatic patients compared to healthy controls [139,140,141,142]. In the microenvironment, IL-33 acts as an alarmin, recruiting the innate immune response cells, such as mast cells [140]. Mast cells in turn can activate neutrophils and can attract keratinocytes, inducing the development of skin inflammation, such as in the psoriatic lesions, through the degranulation and production of various cytokines, including IL-1 and IL-6 [143].

**Figure 2:**
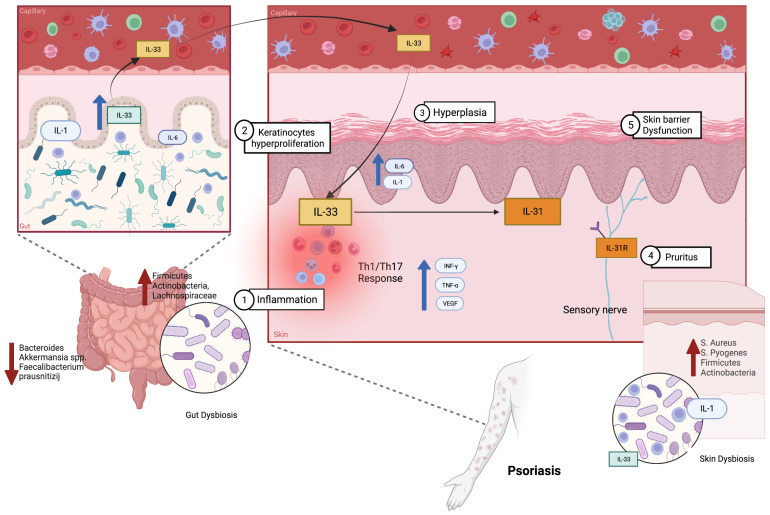
The role of gut and skin dysbiosis and the IL-33/31 axis in inducing psoriatic lesions.

In addition, similarly to the findings in AD, Dual et al. suggest that IL-33 may induce or exacerbate the epithelial hyperplasia, which is the main histological characteristic of psoriatic lesions [144]. In fact, two studies also reported that IL-33 was observed in the nucleus of keratinocytes within the suprabasal layer of the stratum spinosum of psoriatic skin (Figure 2).

The release of IL-33 after skin damage promotes an inflammatory response (characterized by the upregulation of cytokines such as IL-6, IL-20 and MCP-1) and angiogenesis (via VEGF) [145,146,147]. These data are very important because VEGF is implicated in the pathogenesis of psoriasis and it correlates with its clinical severity [30]. Patruno et al. showed that IL-33 also seems to be an inflammatory pain mediator, given that its levels in a skin biopsy positively correlate with the NRS scale and Pain Qualities Assessment Scale (PQAS) [147]. Interestingly, TNFα, INFγ and IL-17A, which are the main effectors of the Th1/Th17 response in psoriasis pathogenesis, were also found to stimulate the release of IL-33 [30]. As already mentioned, IL-33 may active IL-6 and its family, in particular IL-31. Indeed, its levels are also significantly elevated in psoriasis compared to healthy controls [148], which decreased upon UVB irradiation [142]. Nattkemper et al. documented that IL-31 has an increased gene transcript level in itchy psoriatic skin [140]. In addition, IL-31 can be involved in the development of psoriatic lesions due to its possible influence of angiogenesis and chronic inflammation, acting as a proangiogenic factor inducing the expression of VEGF in epithelial cells [149,150].

### 2.3. Confronting Research Results

Satisfying research results are available indicating a pathogenic link between the IL-33/IL-31 axis and psoriasis and between dysbiosis and psoriasis. Still, no study was found discussing the pathogenic link between these three components, unlike for atopic dermatitis. Although Staphylococci are common colonizers found both in AD and psoriasis and the external stimuli triggering these diseases are overall similar, the difference in barrier integrity and keratinocyte signalling machinery is what determines the different inflammatory responses that characterize these diseases [151]. Still, the direct effect of Staphylococcus Aureus observed on the IL-33/IL-31 axis suggests that the findings observed in AD could also be extended to psoriasis. In addition, although there are interesting studies about the linkage between the IL-33/31 axis and dysbiosis, its role still has further investigation potential. We suggest conducting further investigations on the matter.

## 3. Therapeutic Perspectives

### 3.1. Therapeutic Approaches for Dysbiosis in Atopic Dermatitis and Psoriasis

Staphylococcus Aureus and its toxins appear to be ideal targets for management of AD, and plenty of studies have been conducted in this regard (Table 1).

A novel functional ingredient, hyaluronic acid combined with a fragment of Cutibacterium acnes bacterial wall (HAc40), was shown to sequestrate and inactivate *S. aureus* toxins in porcine skin with a post-biotic action in a two-pilot vehicle-controlled trial. The studies conducted demonstrated a direct effect of HAc40 on α-toxin (a crucial virulence factor necessary to impair skin barrier integrity), but Magnifico et al. suggested to further expand the findings by also investigating the effects on δ-toxin and other virulence factors [152]. Another topical agent, a multi-action emollient plus cream (EC; Dermoflan^®^), has been developed as a maintenance therapy for adult patients with mild-to-moderate AD in clinical remission. This EC contains, among other substances, prebiotics that inhibit the growth of harmful bacteria and promote the growth of beneficial bacteria such as Staphylococcus epidermidis to maintain the natural skin microbiome, and it has been demonstrated to promote skin barrier repair and increase levels of toll-like receptor (TLR) [153]. In fact, patients with AD have increased susceptibility to microbial infections, which may be due to abnormalities in the TLR function, which have been linked to a reduced ability to clear staphylococcal infections [154]. By increasing TLR-1 and TLR-2 expression in all epidermal layers, EC treatment may support the epidermal defence against pathogenic penetration [153]. Finally, Di Domenico et al. hypothesized that the inefficacy of antibiotic therapy in the treatment of moderate and severe forms of AD could be explained by the presence of biofilm produced by clonal *S. aureus* and suggested that bacteriotherapy with biofilm-producing skin commensal species competing against *S. aureus* biofilm could be a successful therapeutic strategy [155]. Because AD patients exhibit a decreased production of sebum and its microbial metabolite, propionate, a recent study has shown that topical propionate application attenuates skin inflammation in mice with MC903-induced AD-like dermatitis by inhibiting IL-33 production in keratinocytes. A proof-of-concept clinical study further demonstrated the beneficial therapeutic effects of topical propionate application in mild-to-moderate AD patients, indicating that the sebum–microbial metabolite–IL-33 axis is involved in the pathogenesis of AD, possibly by playing an initiating role in the induction of skin inflammation [119].

Different microbiome-modifying treatments for AD are currently being tested on mice models. In NC/Nga mice, topical application of josamycin ointment inhibits the development of AD-like skin lesions through regulation of *S. aureus* skin colonization and scratching behaviour, associated with the expression of Th2 cytokines and IL-31 mRNA in the lesions [156]. Moreover, some plant extracts such as resveratrol, rice components and P. densiflora bark extract have been shown to reduce IL-31 production topically by downregulating mRNA expression of Th2 and Th17 cytokines [157,158]. Other promising topical agents are the coumarins from the fruit of Cnidium monnieri (TCFC), which upregulated the filaggrin mRNA in the skin of rats and downregulated the levels of IL-1β, IL-4, IL-31 and TSLP mRNA [159].

Oral probiotics can also contribute to preventive and/or therapeutic strategies for AD by modulating the host immune system and the gut microbiota. Laboratory studies have reported that Lactobacillus spp., major probiotics known for their lactic acid production, have strong effects on the host’s immune responses such as decreases in Th1-, Th2- and Th17-related cytokines or increases in IL-10 or CD4+CD25+ regulatory T cells [160,161,162]. Another study on a mouse model showed that oral administration of *Lactobacillus Fermentum* KBL375 showed various protective effects against AD, such as improvements in clinical symptoms, immunomodulation of the host, lowering of serum-immunoglobulin-E level and changes in metabolic pathways due to gut microbiota restoration. This reduction in disease activity came along with decreased levels of IL-4, IL-5, IL-13 and IL-31 and increased levels of anti-inflammatory cytokine IL-10 and transforming-growth factor-β in skin tissues [162]. Oral administration of tyndallized *Lactobacillus rhamnosus* IDCC 3201 (RHT3201) showed significant SCORAD reduction, correlated with a decrement in eosinophil cationic protein and IL-31 [163].

**Table 1:** Novel therapies in atopic dermatitis.

Study	Molecule	Study Population	Samples	Mechanism of Action/Results
Magnifico I. et al. [152] 2023	Hyaluronic acid combined with a fragment of *Cutibacterium acnes* (HAc40)	Pigs	Porcine ear skin explants infected with *S. Aureus*	Prevention and protection of the stratum corneum of tight junction tissues and proteins from the damage of *S. aureus* infection.
Quadri M. et al. [153] 2021	Emollient plus cream containing probiotics (EC: Dermoflan)	Vitro/Humans (20)	Skin biopsies and patients with mild-to-moderate AD	EC once daily for 2 months; increase in epidermal thickness, lipid content and TLR; growth of beneficial bacteria.
Qiu Z. et al. [119] 2022	Topical propionate	Mice	Skin with MC903-induced AD-like dermatitis	Reduction in IL-33 production by keratinocytes and attenuation of skin inflammation.
Matsui K. et al. [156] 2017	Josamycin (0.1%)	Mice	AD-like skin lesions	Regulation of *S. aureus* skin colonization, reduction in IL-31 mRNAs and Th2 expression.
Kang M.C. et al. [157] 2019	Resveratrol-Enriched Rice (RR)	Mice	AD-like skin lesions	Reduction in scratching frequency, dermatitis severity and trans-epidermal water loss (TEWL), decreased IL-6, IL-31 and IgEs serum levels.
Lee J.W. et al. [158] 2019	*Pinus densiflora* bark extract (PBE)	Mice	AD-like skin lesions	Reduction in AD dermatitis scores, scratching behaviour and epidermal thickness; decreased mast cells, eosinophils, IgEs, Th2 and Th17 cytokines serum levels.
Zhijie Y. et al. [159] 2021	Total coumarins from the fructus of *Cnidium monnieri* (TCFC)	Mice	AD-like skin lesions	Reduction in epidermal thickness, decreased mast cells, eosinophils, IgEs, Th2 and Th17 cytokines serum levels.
Kwon M.S. et al. [160] 2018	*Lactobacillus sakei* (WIKIM30)	Mice	AD-like skin lesions	Reduction in skin lesions and decreased CD4+ T-cells and B-cells, IgEs, Th2 cytokines serum levels. Increase in beneficial intestinal bacteria.
Jeong K. et al. [163] 2020	*Lactobacillus rhamnosus* (RHT3201)	Humans (100)	Children (aged 1–12 years) with moderate AD	RHT3201 once daily for 12 weeks; reduction in SCORAD total score, decrement in eosinophil cationic protein and IL-31.

Alterations in the gut and skin microbiomes have been shown to interact with host immunity and affect skin barrier function. Still, the role of antibiotics in the treatment of psoriasis remains controversial and there are several research gaps to be filled. Macrolides and rifampin were shown to improve the PASI score and pruritus in patients with plaque psoriasis, but these results are thought to be the attributed to their immunomodulatory effect [59]. Coal tar is used to treat scalp psoriasis for its antiseptic and anti-inflammatory effects and its ability to improve microbial diversity [164].

Current evidence suggests that modulation of the gut microbiota, both through dietary approaches and through supplementation with probiotics and prebiotics, could represent a novel therapeutic approach in psoriasis (Table 2). Adherence to the Mediterranean diet seems to reduce the severity status of certain dermatological pathologies. Anti-inflammatory effects of this diet could be explained by the high intake of omega 3 fatty acids present in the Mediterranean diet which are linked with favourable outcomes regarding their effects in psoriasis patients [165]. Moreover, the Mediterranean diet could also enrich the gut microbiota diversity, including bacteria with anti-inflammatory properties [166]. Supplementation with *Bifidobacterium infantis* in psoriasis patients for 6–8 weeks resulted in reduced pro-inflammatory status by lowering the plasma CRP and LPS-stimulated TNF-α and IL-6 levels; moreover, a multi-strain probiotic also highlighted the improvement in the PASI (Psoriasis Area Severity Index) score and quality of life among psoriasis patients after two months of supplementation, reducing pro-inflammatory cytokines (hs-CRP and IL1-β) and LPS serum levels [167]. A recent study addressed the effect of quercetin supplementation (30, 60 and 120 mg/kg) on imiquimod-induced mice, showing drastically improved PASI scores; quercetin successfully reduced serum TNF-α, IL-6 and IL-17 levels and strengthened the anti-inflammatory effect [168].

### 3.2. Biologic Therapies targeting the IL-33/31 Axis

As AD and psoriasis became better understood, different molecules have been engineered throughout the last decade for the treatment of these diseases. The therapeutic choice is not only influenced by the patient’s individual preference but also by the endotype of their disease [169]. In the light of the complex immunopathological mechanisms described at the basis of “IL-33/IL-31 axis theory” as well as on the microbiome, being able to intervene in the modulation of this cytokine axis could represent a promising therapeutic perspective for selected patients. Symptoms such as itching, which is a cornerstone of atopic dermatitis and can affect sleep and overall quality of life, can be managed through specific monoclonal antibodies against the mediators involved in this cytokine axis.

To this day, anti-IL-31 agents are approved only for veterinary use. Lokivetmab is a caninized IL-31 monoclonal antibody which was approved in 2017 by the European Medicines Agency for the treatment of canine atopic dermatitis. A two-part phase 1 single-dose trial was conducted between 2012 and 2015 to assess the efficacy of an anti-human IL-31 antibody (BMS-981164) produced by Bristol-Myers Squibb for atopic dermatitis, but the results were never published. Instead, pharmaceutical research is currently focused on molecules blocking the IL-31 receptor. Nemolizumab is an anti-human IL-31RA antibody which is currently being studied for the treatment of pruriginous diseases such as AD, AD-associated pruritus, prurigo nodularis, chronic kidney disease-associated pruritus and systemic sclerosis. It was approved in Japan in 2022 for the treatment of itching associated with atopic dermatitis after it was shown to be able to significantly improve pruritus severity scores when used in addition to topical agents [170,171]. Phase 3 trial results regarding its efficacy on the severity of eczema in atopic dermatitis (ARCADIA 1 and 2 (NCT03989349, NCT03989206)) and nodules in prurigo nodularis (OLYMPIA 1 and 2 (NCT04501666, NCT04501679)) are expected to be published in 2023, and nemolizumab is planned to be launched in the U.S. in 2024 [172]. It is not currently being investigated for the treatment of psoriasis, but if the role of IL-31 in the pathogenesis of this disease becomes more established, nemolizumab could also be evaluated as a therapeutic option for the treatment of psoriasis-associated pruritus. Instead, vixarelimab is a human anti-OSMRβ antibody, which is currently being studied for the treatment of pruriginous diseases. Recently, results from a randomized, double-blind, placebo-controlled phase 2a trial for the treatment of moderate-to-severe prurigo nodularis were published [173], while data from an exploratory phase 2 study (NCT03858634) for the treatment of other diseases characterized by chronic pruritus (including plaque psoriasis) were released in 2020 [174], but no peer-reviewed publication is available yet.

Results for biological drugs targeting IL-33 have been less promising. Etokimab (ANB020) is an anti-IL-33 humanized monoclonal antibody, which was assessed in adults with moderate-to-severe atopic dermatitis. A Phase IIa proof-of-concept clinical trial showed improvement in symptoms of atopic dermatitis and reduced skin neutrophil infiltration as well as peripheral eosinophil counts after a single systemic administration of etokimab, [175] but in November 2019, it was announced that the phase 2b trial (NCT03533751) failed to meet its primary endpoint (reduction in EASI relative to placebo at week 16) [176]. Similarly, astegolimab was an anti-ST2 antibody which was being investigated for the treatment of moderate-to-severe AD, but it failed to meet its phase 2 trial primary endpoint (reduction in EASI relative to placebo at week 16) [177]. Itepekimab (REGN3500) is another anti-IL-33 monoclonal antibody which was investigated for the treatment of AD as a monotherapy and in combination with dupilumab, but both trials (NCT03738423, NCT03736967) were terminated due to lack of efficacy. Melrilimab (CNTO7160 / GSK-3772847) is a monoclonal antibody targeting IL-33R which underwent a phase I clinical trial in patients with asthma, AD and healthy individuals. Safety data were published in 2020, showing effective inhibition of the IL-33R signalling pathway, although this did not translate into significant clinical improvement [178], and phase 2 trials have been carried out only for asthma [179]. Finally, PF-06817024 is one of the most recent anti-IL-33 monoclonal antibodies developed by Pfizer; a phase I placebo-controlled trial in healthy subjects, patients with chronic nasal sinusitis and patients with AD was completed in 2022, with no evidence of serious adverse events (NCT02743871) [180].

## 4. Conclusions

AD and PsO are chronic inflammatory skin diseases that manifest clinically in different ways. However, these pathologies share immunopathogenetic mechanisms underlying their inflammation with an overexpression of IL-31 and IL-33. These two interleukins cooperate with each other up to the theory definition of the IL-33/IL-31 axis, precisely because the presence of one interleukin can stimulate the induction of the other. In fact, the expression of IL-33 signalling through the NFkB pathway induces the release of IL-31, just as IL-4 enhanced by IL-33 induces the gene expression of IL-31. Furthermore, it is very interesting how it has been clinically demonstrated that the severity of the disease in terms of skin lesions and disease symptoms is associated with serum and tissue expression of IL-31 and IL-33 levels, demonstrating how there is a close correlation between these two cytokines in these inflammatory pathologies. It has been described that both AD and PsO are characterized by microbial alterations and the role of skin and intestinal dysbiosis has been extensively studied in recent years. There are so many therapeutic approaches that by altering the composition and function of the microbiome they can be used as preventive or therapeutic strategies in the management of atopic dermatitis and psoriasis. In particular, the link between dysbiosis and IL-33/IL-31 in AD and PsO support the concept that dysbiosis may act as an aetiological culprit in the development of skin conditions through immune dysregulation.

## Figures and Tables

**Table 2:** Novel therapies in Psoriasis.

Study	Molecule	Study Population	Samples	Mechanism of Action/Results
Thami G. et al. [165] 2002	Coal tar	Humans	Scalp psoriasis	Anti-septic and anti-inflammatory role.
Moludi J. et al. [167] 2022	*Bifidobacterium Infantis*	Humans	Psoriasis Skin	Supplementation for 6–8 weeks; reduction in CRP and LPS-stimulated TNF-α and IL-6 level; improvement in PASI and DLQI score.
Chen H. et al. [168] 2017	Quercitin (QC)	Mice	Imiquimod-induced psoriasis-like mouse model	Reduction in PASI score and TNF-α, IL-6 and IL-17 serum levels.

## Data Availability

No new data were created or analyzed in this study. Data sharing is not applicable to this article.
